# Survival of 2nd trimester pregnant patient and di-di twin on COVID-19 ECMO

**DOI:** 10.1051/ject/2023012

**Published:** 2023-06-28

**Authors:** Yookwi Lee, Kholoud Nassar, Atit Parikh

**Affiliations:** 1 Loma Linda University Medical Cener Loma Linda California

**Keywords:** ECMO, COVID-19, Pregnancy

## Abstract

A 35-year-old unvaccinated woman, pregnant with twins at 22 weeks and 5 days of gestation presented with worsening hypoxia, due to COVID-19 pneumonia (PNA) with acute respiratory distress syndrome (ARDS). The patient was placed on V-V ECMO (veno-venous extracorporeal membrane oxygenation) and delivered twin babies by cesarean section (C-section) at 23 weeks and 5 days of gestation. The patient was successfully weaned off ECMO 42 days after initiation, and the twins were also extubated in NICU.

Extracorporeal membrane oxygenation can provide respiratory support or both respiratory and circulatory support. V-V ECMO is specifically utilized to provide respiratory support for patients who do not respond to conventional treatment. Furthermore, V-V ECMO reduces the onset of ventilator-induced lung injury (VILI). Although pregnancy is not an absolute contraindication for ECMO, careful patient selection is recommended to reduce the risk of maternal and fetal complications. Limited literature exists on ECMO initiation and management for the pregnant patient subgroup. This is a single-center report to detail the procedures that rendered the successful use of V-V ECMO on a pregnant patient who developed COVID-19 PNA with ARDS.

## Description

A 35-year-old woman with dichorionic diamniotic twin (di-di twin) pregnancy (22 weeks and 5 days) was transferred to Loma Linda University Medical Center for COVID-19 PNA with ARDS. Per previous hospital records, the patient tested positive for COVID-19 and had an elevated beta-hydroxybutarate, anion gap, which required oxygen due to increasing tachypnea. She was medicated with Remdesivir, Dexamethasone, Azithromycin, and Rocephin. She had gestational diabetes mellitus. She was intubated immediately prior to transfer due to worsening tachypnea which required high flow nasal cannula (HFNC) and bilevel positive airway pressure (BiPAP). The indication for V-V ECMO was worsened PNA with intubation and subsequently worsening ARDS with maximal medical therapy.

Prior to ECMO cannulation, the patient’s baseline ACT (Hemochron) was checked, and a half-standard dose (50 units/kg) of 2,000 units of heparin were given. The ECMO circuit (CardioHelp) was carefully warmed to 37 °C and maintained at normothermia to minimize the risk of decreasing fetal heart rate. The patient was paralyzed and sedated with Nimbex, Fentanyl, and Propofol. A 27Fr multistage venous cannula (Medtronic) and a 20Fr Fem-Flex arterial cannula (Edwards) were placed in the left femoral vein and right internal jugular vein respectively. After measuring ACT of 170 s, V-V ECMO was initiated with a pump flow of a Cardiac Index (CI) of 3.0 L/m^2^/min. The patient’s saturation and acidosis were improved. The ventilator setting was readjusted to AC/PC mode, RR 12, PIP/PEEP 20/10, FiO_2_ 40% with 20 ppm of inhaled nitric oxide (iNO). ACT goal was set to 130–150 s and checked every hour. The target hemoglobin was >10 g/dL, and a pump flow goal of CI of 2.8–3.0 L/m^2^/min was established to compensate for the low ACT target range along with expected high oxygen consumption. The flow rate was adjusted based on the patient’s arterial oxygen saturation. Pre/post oxygenator blood gases, venous inlet pressure, pre/post oxygenator pressures, and transmembrane pressure (delta P) were measured every 6–8 h as per standard protocol. Doppler tones were scheduled every 3 days. On day 2 of ECMO, a 10 unit/kg/h heparin drip was initiated, which was lower than the standard dosage of 12 unit/kg/h. ACT was checked every hour for the first 24 h and every 2 h during the subsequent days. CBC, electrolytes, BMP, liver enzymes, renal function and blood sugar were quantified every 6–8 h, per standard protocol. Additionally, platelet count and plasma-free Hgb were collected twice a day. Daily INR, PT, aPTT (every 6 h), fibrinogen anti-Xa, stat functional ATIII and d-dimer were measured to monitor adequate anticoagulation. The paralytic was discontinued, and the iNO setting was reduced to 10 ppm. On day 3 of ECMO the patient developed right intraparenchymal hemorrhage (IPH) and subarachnoid hemorrhage (SAH), leading to the hold of anticoagulation. While the heparin drip was being held, ACT and aPTT were measured to assure the circuit integrity every 4 h and 6 h respectively. Keppra was given as a seizure prophylaxis and iNO was disconnected from the ventilator. On day 5 of ECMO, due to her persistent hypertension, preeclampsia was suspected, and a lab test was ordered. On day 6 of ECMO, the anesthetic was held. The patient opened her eyes but did not move her extremities. After a head CT showed no interval change. The patient was paralyzed to further reduce oxygen consumption.

Despite ECMO support, the patient’s hypoxia was worsening and her overnight PaO_2_ level dropped as low as 40 mmHg. A daily chest X-ray confirmed appropriate cannula positions and ensured proper circulation. Based on OB consultation, maternal oxygenation could be improved by up to 10% in the given gestational age by delivering the babies. Furthermore, fetal middle cerebral arterial (MCA) dopplers suggested possible inadequate fetal oxygenation, however, viable weight for delivery was confirmed. After a multidisciplinary family meeting, on day 7 of ECMO, 23 weeks and 5 days gestation, a C-section was performed under general anesthesia at the bedside. Both twins were delivered with no complications and were noted to have fetal movements. During the C-section, an ECMO flow CI of 2.8 L/m^2^/min was maintained. Intraoperative blood loss was approximately 900 mL. Two units of RBC and two units of FFP were transfused through the ECMO circuit intraoperatively. The patient was extubated on day 11 of ECMO and put on HFNC. Anticoagulation was restarted and converted to a Bivalirudin drip of 0.1 mg/kg/h on day 12 of ECMO. Bivalirudin titration was adjusted by 0.005 mg/kg/h per standard protocol. aPTT was monitored every 3 h for the first 24 h and every 6 h subsequently, along with INR, PT, and platelet count. Daily fibrinogen and d-dimer counts were collected. The patient began tilting bed sessions with physical therapists. The patient’s mental status showed improvement on day 16 of ECMO. On day 38 of ECMO, the patient ambulated in the CT ICU hallway. The next day, the ECMO sweep was turned off. Forthwith, the patient was successfully decannulated from V-V ECMO 42 days after initiation. During ECMO support, the ECMO circuit was changed once on day 22 due to progressively reduced oxygenator efficiency (post oxygenator PaO_2_: 160 mmHg; Patient SpO_2_: 83.3%) and hemolysis (PFHb: 194.7 mg/dL) accompanied by cumulative fibrin deposits and oxygenator thrombosis. She had three episodes of atrial fibrillation (af) with rapid ventricular response (RVR), but sinus rhythm (SR) was restored with Amiodarone. Anticoagulation was held intermittently due to epistaxis and melena postpartum. The twins were both extubated and doing well in the NICU at the time of submission. The patient was routinely turned, but she was not particularly placed in the left lateral tilt (LLT) position due to the early stage of the pregnancy. Also, ECMO venous drainage was undisturbed and adequate perfusion to the fetuses was confirmed.

## Comment

The impact of COVID-19 infections on pregnant patients and the care of COVID-19 compromised pregnant patients on ECMO are novel arenas. Management of pregnant COVID-19 patients with an ECMO circuit is challenging due to their unique physiology and the characteristics of the virus. A great deal of careful consideration is needed to optimize the treatment of these patients. During the 21–24 weeks of gestation, the maternal blood volume, cardiac output, and oxygen consumption increase by 50%, 30–50%, and 20–30% respectively [1]. To accommodate these changes, ECMO flow rates and patient hemoglobin need to be adjusted to higher ranges (2.8–3.0 L/m^2^/min and >10 g/dL) than non-pregnant patients (2.4–2.8 L/m^2^/min and 8–10 g/dL) to provide adequate oxygen delivery. Although mild cooling (35–35.5 °C) techniques are often utilized to increase oxygen saturation during ECMO, it is unknown what degrees of cooling would be harmful to fetuses, therefore cooling of this patient subgroup should be avoided to prevent the decrease of the fetal heart rate and inducing uterine contraction [1]. Due to the large molecular weight restricting transmission across the placental barrier [1], heparin was the safest drug for anticoagulation. The patient was anticoagulated by Bivalirudin (0.1 mg/kg/h) postpartum as per LLUMC V-V ECMO protocol. Balancing adequate and safe anticoagulation is a constant challenge for ECMO procedures. Physiological augmentations of this patient subgroup create multiple considerations. Along with increases in blood volume, there may also be an increase in coagulation factors which possibly require higher levels of heparin dosage, possibly resulting in placental hemorrhage [1]. In addition, COVID-19 patients commonly exhibit issues with hypercoagulability associated with profound inflammatory responses [2]. Therefore, reduced ACT parameters (130–150 s) were maintained by carefully monitoring aPTT, anti-Xa, platelet, stat function ATIII, fibrinogen, and d-dimer to prevent excess heparinization prepartum. Postpartum anticoagulation was switched to standard LLUMC V-V ECMO bleeding protocol (aPTT: 50–70 s and ACT: 160–180 s).

Although the patient and her twin dyad survived this treatment, we endeavor to improve the practice and the protocols to manage maternal hypoxia on ECMO for the early gestation period by reassessing this case. Despite improvements in patient oxygen saturation, the improvement was not calculated due to the inability to acquire accurate patient weight. As an additional note, we observed advanced lung development in the twins for their gestational age (younger than 24 weeks of gestation). From a clinical perspective, gestation of less than 23 weeks is considered a nonviable pregnancy [3]. Therefore, we hypothesized that the maternal steroid treatment for her COVID-19 infection (Dexamethasone and Methylprednisolone) could have accelerated fetal lung development, although research suggests the steroid effect for preterm lung development takes place if it is used between 23 to 33 weeks of pregnancy [4].

V-V ECMO has been an extremely useful device to support patients with respiratory distress and respiratory failure. Data for ECMO treatment on pregnant patients is still limited. However, according to recent reports, the maternal and fetal survival rates on ECMO support during pregnancy were 75.4% and 64.7% [5]. Overall, in-hospital mortality for patients with COVID-19 and ARDS that received ECMO treatment was 49.5% according to a recent large study conducted by Nguyen et al. [6]. As Barrantes et al. state those pregnant patients are younger, healthier, and optimally managed compared to the general population [7]. Therefore, with or without COVID-19, by careful patient selection and additional consideration of normal ECMO protocol, the survival rates for pregnant patient populations could be much higher than the general population.

## Data Availability

All available data are incorporated into the article.
